# Inhibition of EZH2 triggers the tumor suppressive miR-29b network in multiple myeloma

**DOI:** 10.18632/oncotarget.22507

**Published:** 2017-11-20

**Authors:** Maria Angelica Stamato, Giada Juli, Enrica Romeo, Domenica Ronchetti, Mariamena Arbitrio, Daniele Caracciolo, Antonino Neri, Pierosandro Tagliaferri, Pierfrancesco Tassone, Nicola Amodio

**Affiliations:** ^1^ Department of Experimental and Clinical Medicine, Magna Graecia University of Catanzaro, Catanzaro, Italy; ^2^ Department of Oncology and Hemato-oncology, University of Milan, Milan, Italy; ^3^ Hematology, Fondazione IRCCS Ca' Granda Ospedale Maggiore Policlinico, Milan, Italy; ^4^ ISN-CNR, Roccelletta di Borgia, Catanzaro, Italy; ^5^ Sbarro Institute for Cancer Research and Molecular Medicine, Center for Biotechnology, College of Science and Technology, Temple University, Philadelphia, PA, US

**Keywords:** miR-29b, microRNA, miRNA, multiple myeloma, EZH2

## Abstract

Downregulation of tumor suppressor (TS) microRNAs (miRNAs) commonly occurs in human cancer, including multiple myeloma (MM). We previously demonstrated that miR-29b is a relevant TS miRNA, whose expression in MM cells is inhibited by HDAC4-dependent deacetylation. Here, we provide novel insights into epigenetic mechanisms suppressing miR-29b in MM. In MM patient-derived plasma cells, we found inverse correlation between miR-29b and EZH2 mRNA expression. Both siRNAs and pharmacologic inhibitors of EZH2 led to miR-29b upregulation, and this effect was ascribed to reduced H3K27-trimethylation (H3K27me3) of miR-29a/b-1 promoter regions. Induction of miR-29b upon EZH2 inhibition occurred together with downregulation of major miR-29b pro-survival targets, such as SP1, MCL-1 and CDK6. Knock-down of the EZH2-interacting long non-coding RNA MALAT1 also reduced H3K27me3 of miR-29a/b-1 promoter, along with induction of miR-29b and downregulation of miR-29b targets. Importantly, inhibition of miR-29b by antagomiRs dramatically reduced *in vitro* anti-MM activity of small molecule EZH2-inhibitors, indicating that functional miR-29b is crucial for the activity of these compounds. Altogether, these results disclose novel epigenetic alterations contributing to the suppression of miR-29b molecular network, which can be instrumental for the development of rationally designed miRNA-based anti-MM therapeutics.

## INTRODUCTION

Multiple myeloma (MM) is the second most common hematological cancer worldwide and is characterized by progressive accumulation of tumor plasma cells (PC) in the bone marrow (BM), paraprotein production, renal failure and bone destruction [1].

Research carried out in the last decade has disclosed an unprecedented role of the non-coding genome in PC biology, as well as its relevant contribution to cell transformation in the context of PC dyscrasias [2–4]. Among non-coding RNAs, to date the best characterized class is represented by microRNAs (miRNAs), short non-coding RNAs that post-transcriptionally regulate gene expression by binding to the 3′ UTR of mRNA targets, then triggering mRNA and/or protein degradation [2, 5]. Notably, miRNAs can regulate several signal transduction pathways involved in MM pathogenesis [6, 7]. Depending on their mRNA targets, miRNAs can act either as oncogenes [8–13] or as tumor suppressor (TS) miRNAs [14–16], which finely tune the interaction between MM cells and the BM *milieu* [3, 17]. Among TS miRNAs, our group and others demonstrated that miR-29b is a relevant anti-cancer miRNA in a wide variety of solid and hematologic malignancies [18]. In MM cells, ectopic miR-29b was shown to downregulate major tumor promoting or anti-apoptotic mRNA targets, including CDK6, MCL-1, SP1 [14], as well as mRNAs coding for epigenetic regulators, such as HDAC4 [19] and DNMT3A/B [20], thus triggering cell cycle arrest and apotosis.

The comprehension of cancer-related mechanisms involved in downregulation of miR-29b is nowadays a matter of intense investigation, in order to rationally design new therapeutic tools restoring the expression of this relevant TS miRNA. In this regard, it has been demonstrated by us and others that genetic and epigenetic aberrations drive the silencing of miR-29b in hematological malignancies, such as MM and acute myeloid leukemia (AML) [18]. In the context of epigenetic alterations, aberrant deacetylation of miR-29a/b-1 promoter by histone deacetylases (HDACs), such as HDAC1, HDAC3 [21] and HDAC4 [19] represents a well-documented mechanism by which tumor cells silence miR-29b; consistently, pan HDAC-inhibitors have been found to upregulate miR-29b expression in MM [19], AML [21] and CLL [22].

Overexpression of methyltransferases in MM can drive malignant transformation through the silencing of TS genes/non-coding RNAs; in detail, trimethylation of histone H3 at lysine 27 (H3K27me3) or lysine 36 (H3K36me3), catalyzed by the methyltransferases EZH2 and MMSET respectively, leads to the silencing of established tumor suppressor miRNAs [23, 24].

Here, we aimed at identifying novel epigenetic mechanisms regulating miR-29b expression. Our results underscore, for the first time, the role of the H3K27 methyltransferase EZH2 in the negative regulation of miR-29b in MM.

## RESULTS

### miR-29b and EZH2 mRNA expression inversely correlates in primary MM PCs

**Figure 1:**
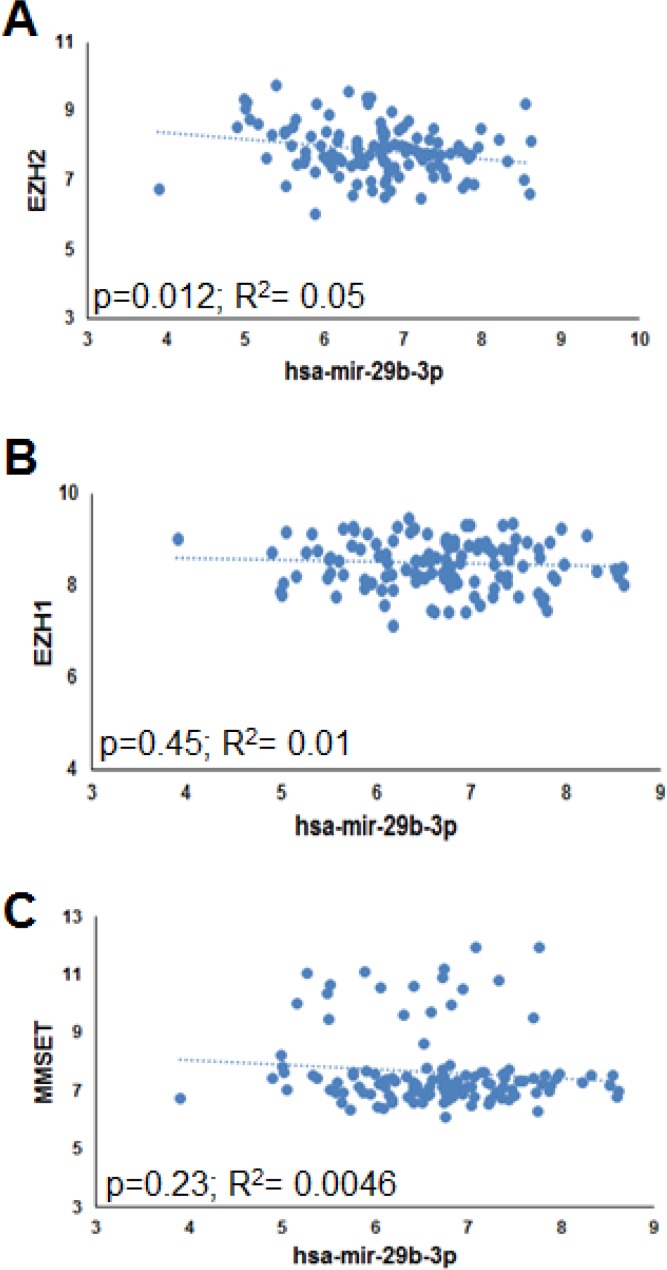
Inverse correlation between EZH2 and miR-29b in MM patient-derived plasma cells Correlation of endogenous miR-29b levels with EZH2 (**A**), EZH1 (**B**) and MMSET (**C**) mRNA levels, determined by high density microarray analysis of mRNA or miRNA expression in GSE73454 (for miR-29b) and GSE73452 (for EZH2 mRNA) datasets. Log values of raw data are reported in graph. R = regression coefficient.

In an attempt to identify novel epigenetic regulators contributing to the silencing of TS miR-29b in MM, firstly we evaluated the potential correlation between miR-29b and the mRNA expression levels of histone methyltransferases with an oncogenic role in MM [23], such as EZH1, EZH2 and MMSET. To this aim, we interrogated proprietary GEP and miRNA datasets obtained from 95 MM and 29 PC leukemia patient-derived PCs. Interestingly, a statistically significant inverse correlation could be observed only between miR-29b and EZH2 (Figure 1A–1C), thus prompting us to investigate the potential role of EZH2 on miR-29b regulation.

### Inhibition of EZH2 promotes miR-29b expression and reduces H3K27me3 marks at miR-29a/b-1 promoter

**Figure 2:**
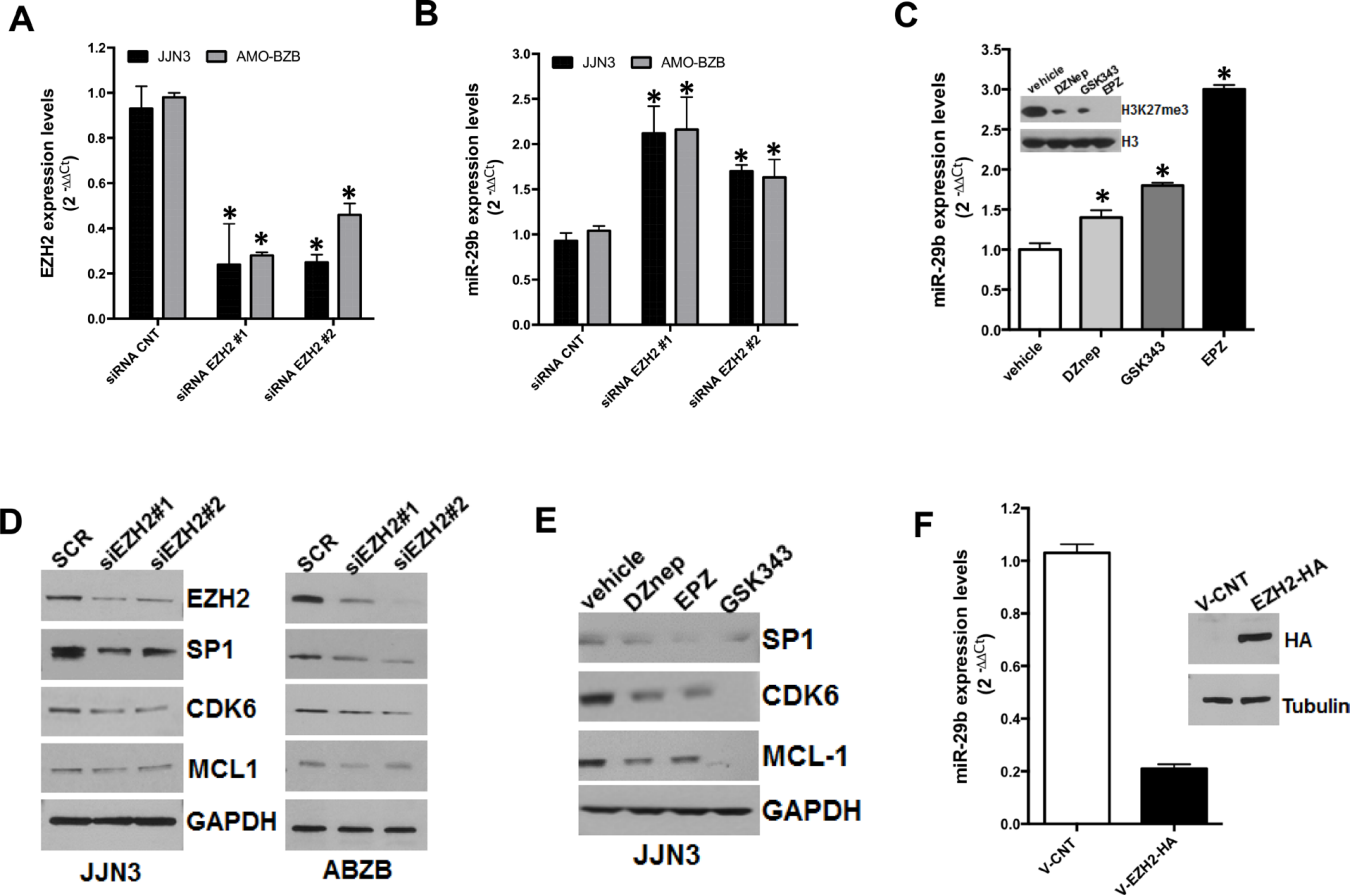
Inhibitory effect of EZH2 on miR-29b expression QRT-PCR analysis of EZH2 (**A**) and miR-29b (**B**) expression levels in AMO-BZB and JJN3 cells, 24 hours after transfection with 100 nM scrambled siRNAs (SCR) or EZH2-targeting siRNAs (siEZH2#1 and siEZH2#2). (**C**) QRT-PCR of miR-29b levels, 24 hours after treatment of JJN3 with 2 μM DZnep, 5 μM GSK343 or 5 μM EPZ005687; WB shows the levels of H3K27me3 and total histone H3 in JJN3-treated cells. (**D**) WB analysis of SP1, CDK6 and MCL-1, 24 hours after transfection of JJN3 or AMO-BZB cells with 100 nM scrambled siRNAs (SCR) or EZH2-targeting siRNAs (siEZH2#1 and siEZH2#2); GAPDH was used as loading control. (**E**) WB analysis of SP1, CDK6 and MCL-1, 24 hours after treatment of JJN3 with 2 μM DZnep, 5 μM GSK343 or 5 μM EPZ005687; GAPDH was used as loading control. (**F**) QRT-PCR analysis of miR-29b expression in AMO-BZB cells, 24 hours after transfection with 2.5 μg of pEZ-M06-EZH2 plasmid (V-EZH2) or of the empty vector (V-CNT). The blot shows levels of EZH2 in AMO-BZB cells, 24 hours after transfection. ^*^P < 0.01.

To investigate the effects of EZH2 on miR-29b expression, we analyzed miR-29b levels in JJN3 and AMO-BZB MM cell lines transfected with scrambled siRNAs (as control) or two different EZH2-targeting siRNAs. QRT-PCR analysis indicated downregulation of EZH2 mRNA transcript (Figure 2A) and upregulation of miR-29b (Figure 2B) as early as 24 hours after EZH2 silencing. Moreover, treatment of MM cells with EZH2 inhibitors, such as the S-adenosyl-homocysteine hydrolase inhibitor 3-Deazaneplanocin (DZNep), GSK343 or EPZ005687 [25, 26], reduced H3K27me3 levels and triggered miR-29b upregulation in MM cell lines (Figure 2C).

We previously showed that miR-29b acts as TS miRNA in MM by targeting cell cycle regulators and anti-apoptotic genes [14]. WB analysis indicated that transfection of EZH2-targeting siRNAs in JJN3 and AMO-BZB cells triggered downregulation of validated miR-29b targets, such as SP1, CDK6, and to a lesser extent MCL-1 (Figure 2D), indicating that EZH2-depletion induced a functionally active miR-29b; downregulation of SP1, CDK6 and MCL-1 was also achieved after treatment of MM cells with EZH2 inhibitors (Figure 2E). Conversely, ectopic expression of EZH2 in AMO-BZB cells resulted in downregulation of miR-29b (Figure 2F). Altogether, these results support a negative role of EZH2 on miR-29b expression and activity.

**Figure 3:**
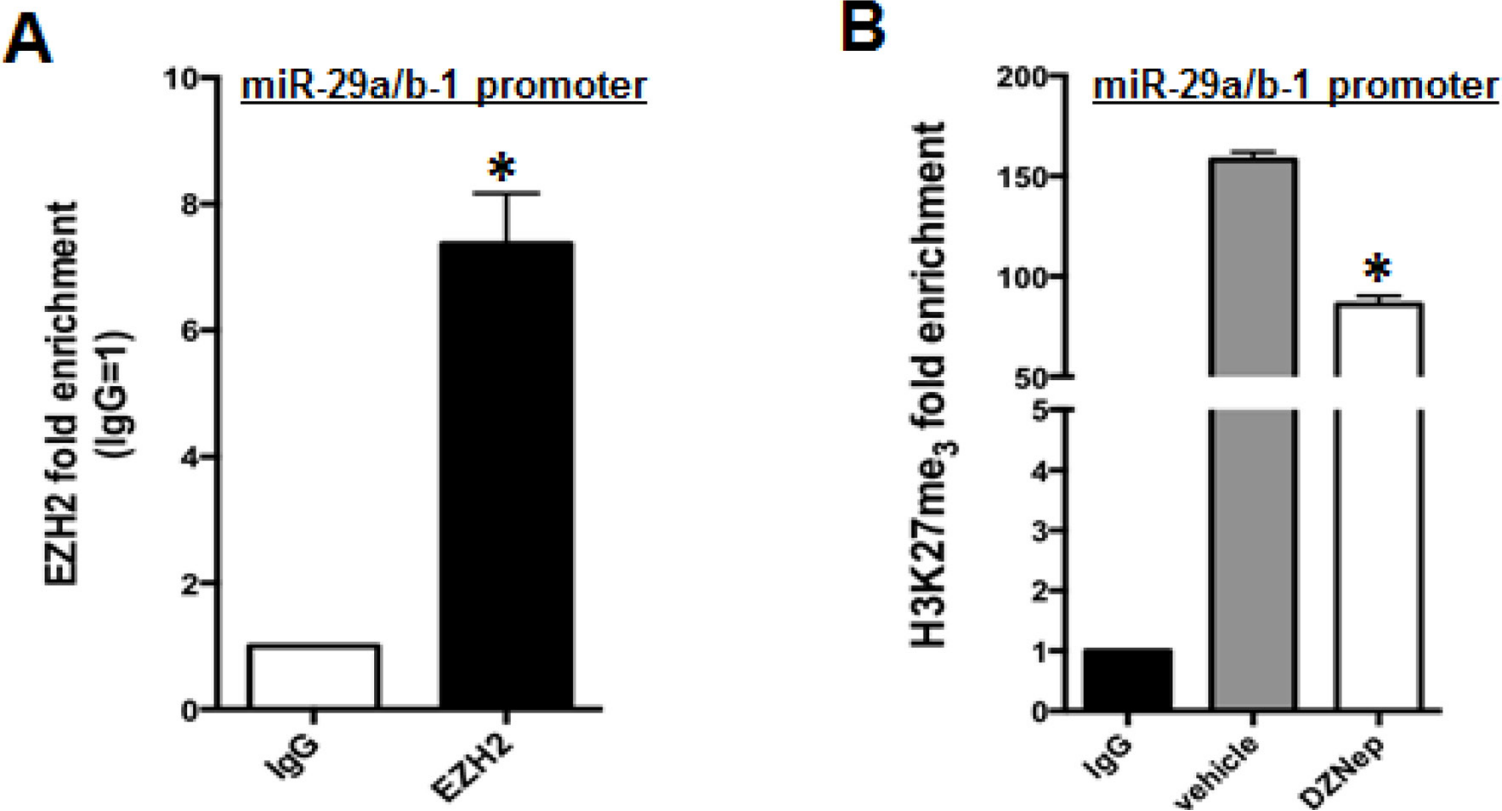
EZH2 binds miR-29a/b-1 promoter and regulates its H3K27me3 (**A**) Chip assay using an EZH2 antibody, or IgG isotypic control, was performed in AMO-BZB cells. Results are the average of three independent experiments performed in triplicate and show enrichment of EZH2 at miR-29a/b-1 promoter regions. ^*^P < 0.01. (**B**) Chip assay using an H3K27me3 antibody or IgG isotypic control, in AMO-BZB cells treated with DMSO or 2 μM DZNep for 24 hours. Results are the average of three independent experiments performed in triplicate and show reduction of H3K27 trimethylation at miR-29a/b-1 promoter regions after inhibition of EZH2 with DZNep. ^*^P < 0.01 respect to DMSO-treated cells.

EZH2 is a methyltransferase whose oncogenic activity relies on promoter H3K27me3 of TS genes and miRNAs, which leads to their transcriptional silencing [23, 27]. On this basis, we sought to evaluate whether EZH2 could bind and affect the H3K27me3 status of miR-29a/b-1 promoter. By using an EZH2 antibody, we performed a ChiP assay on AMO-BZB cells: results indicate a 7-fold enrichment of miR-29a/b-1 promoter sequences in EZH2-immunoprecipitates, as compared with control IgG-immunoprecipitates (Figure 3A), indicating that EZH2 specifically binds to miR-29a/b-1 promoter regions. To verify whether miR-29b upregulation induced by EZH2 silencing could depend on decreased promoter-associated H3K27me3, we analyzed H3K27me3 status of miR-29a/b-1 promoter by Chip. As expected, pharmacologic inhibition of EZH2 by DZNep decreased H3K27me3 at miR-29a/b-1 promoter (Figure 3B), thus suggesting that miR-29b induction occurs by reduced H3K27me3 repressive marks.

### Knock-down of the lncRNA MALAT1 induces expression of miR-29b in MM cells

**Figure 4:**
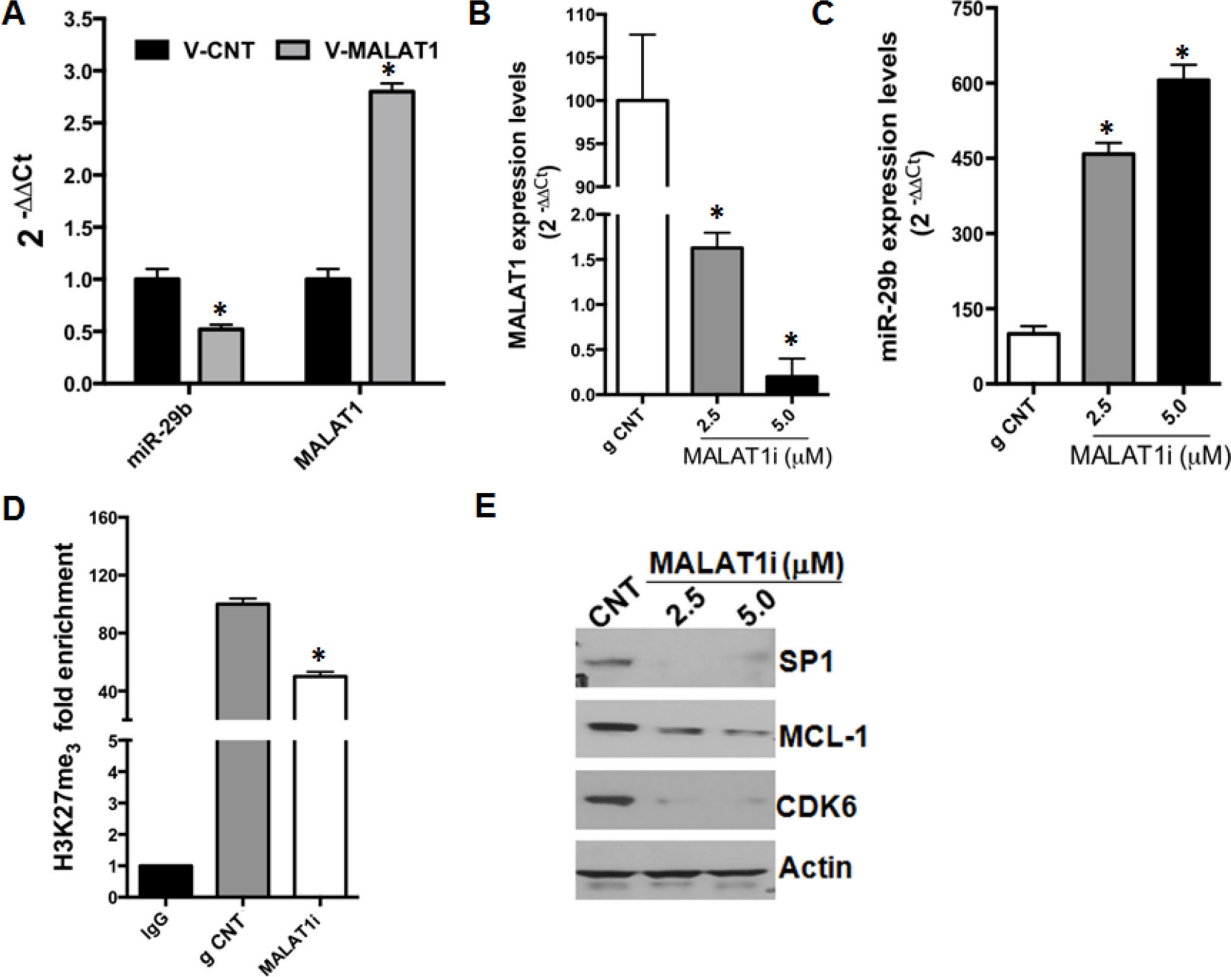
MALAT1 knock-down upregulates miR-29b expression (**A**) QRT-PCR analysis of miR-29b and MALAT1 in AMO-BZB cells after transduction with an empty vector (V-CNT) or a vector containing MALAT1 cDNA (V-MALAT1). QRT-PCR analysis of MALAT1 (**B**) or miR-29b (**C**) expression levels, 72 hours after treatment of AMO-BZB cells with 2.5 μM naked MALAT1 ASOs at the indicated concentrations. ^*^P < 0.01. (**D**) Chip assay using an H3K27me3 antibody or IgG isotypic control, 72 hours after treatment of AMO-BZB cells with naked MALAT1 ASOs. Results are the average of three independent experiments performed in triplicate and show reduction of H3K27 trimethylation at miR-29a/b-1 promoter regions after MALAT1 knock-down with ASOs. ^*^P < 0.01 respect to ASO-control treated cells. (**E**) WB analysis of SP1, CDK6 and MCL-1, 72 hours after treatment of AMO-BZB cells with naked MALAT1 ASOs or control ASOs. GAPDH was used as loading control.

Recent reports have shown that the long non coding RNA (lncRNA) MALAT1 interacts with EZH2 and positively regulates H3K27me3 repressive marks on target gene loci; furthermore, inhibition of MALAT1 was shown to abrogate or reduce EZH2-dependent silencing of target genes [28–30]. To assess whether modulation of MALAT1 could affect miR-29b expression, we used both gain and loss of function experimental strategies. Firstly, AMO-BZB cells were transduced with lentivirus expressing MALAT1, and levels of miR-29b were determined by qRT-PCR. Interestingly, MALAT1 overexpression resulted in downregulation of miR-29b levels in MM cells (Figure 4A). We further analyzed miR-29b levels in AMO-BZB cells treated with selective anti-MALAT1 antisense oligonucleotides (ASOs) [31], that trigger selective RNAse-H dependent degradation of MALAT1, or with a scrambled control. As expected, MALAT1-targeting ASOs strongly downregulated MALAT1 expression, while upregulated miR-29b (Figure 4B–4C). To understand if the upregulation of miR-29b upon MALAT1 inhibition was due to reduced H3K27me3 marks at its promoter, we performed ChiP assay with an H3K27me3 antibody: reduced H3K27 trimethylation of miR-29a/b-1 promoter could be observed in MALAT1-depleted cells, as compared with control (Figure 4D). Similarly to EZH2-targeting siRNAs, anti-MALAT1 ASOs reduced protein levels of validated miR-29b targets, such as SP1, MCL-1 and CDK6 in MM cells (Figure 4E). These results indicate that the lncRNA MALAT1 transcriptionally inhibits miR-29b expression by modulating the amount of H3K27me3 of its promoter.

### Inhibition of miR-29b abrogates *in vitro* anti-MM activity of EZH2 inhibitors

**Figure 5:**
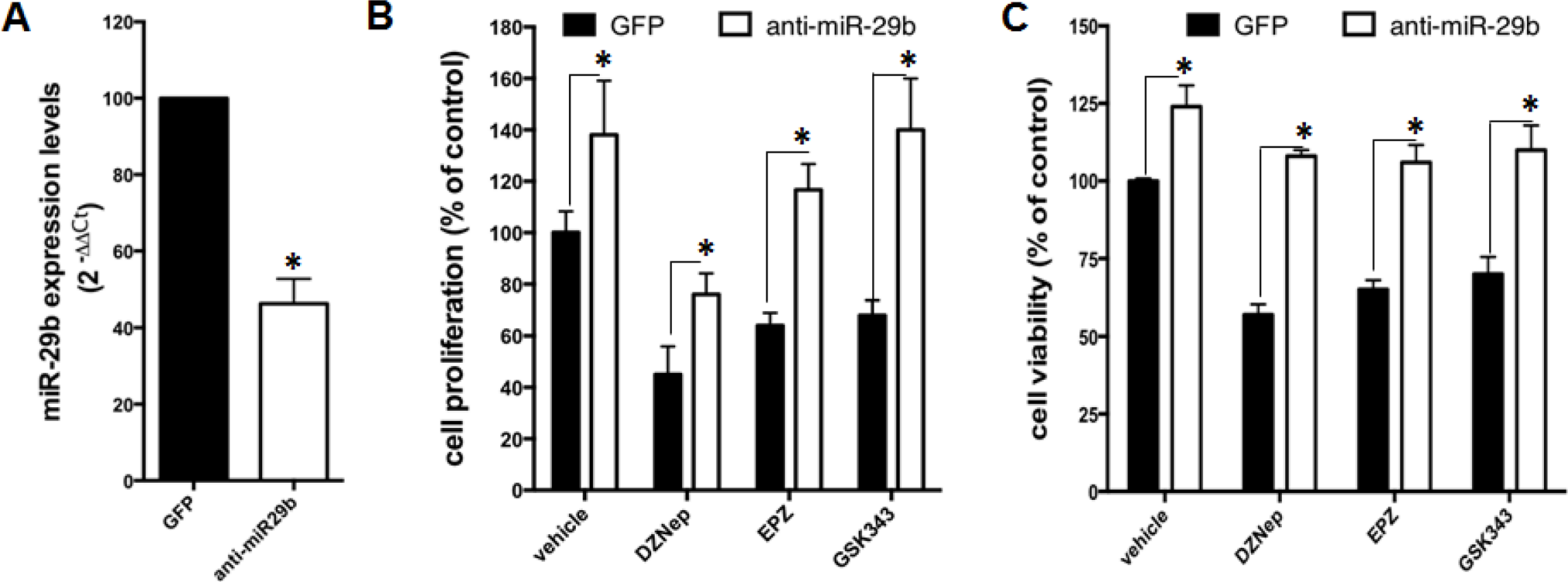
miR-29b antagonism impairs *in vitro* anti-MM activity of EZH2 inhibitors (**A**) QRT-PCR analysis of miR-29b expression levels in AMO-BZB transduced with GFP control or miR-29b inhibitors (antimiR-29b). Trypan blue exclusion assay (**B**) and CTG viability assay (**C**) were performed in AMO-BZB cells stably transduced with GFP control or miR-29b inhibitors (anti-miR-29b), 72 hours after treatment with 2 μM DZnep, 5 μM GSK343 or 5 μM EPZ005687. ^*^P < 0.05.

Small molecule EZH2 inhibitors induce cell cycle arrest and apoptosis, and have demonstrated relevant anti-tumor activity in preclinical models of MM [26]. Since our results show upregulation of miR-29b upon EZH2 inhibition, we asked whether sensitivity of MM cells to EZH2 inhibitors could rely on miR-29b induction. AMO-BZB cells were transduced with specific miR-29b inhibitors (levels of miR-29b are reported in Figure 5A), and then treated with DZNep, EPZ005687 and GSK343. Intriguingly, miR-29b antagonism abrogated the inhibitory effects of these compounds on cell proliferation (Figure 5B) and cell viability (Figure 5C), thus suggesting that miR-29b is crucial in mediating EZH2 inhibitors’ activity.

## DISCUSSION

Substantial progresses have been made in the comprehension of MM pathobiology, which have translated into novel therapeutics, such as proteasome inhibitors, immunomodulatory drugs and novel monoclonal antibodies, with a significant survival advantage for MM patients [1]. There is now compelling evidence that, along with protein-coding genes, the non-coding genome is aberrantly expressed or dysregulated in cancers, including MM [23, 32]. Among the different classes of non-coding RNAs, miRNAs have been extensively investigated for their oncogenic or tumor suppressive role. In the last decade, emerging findings have provided a robust framework for developing miRNA-based therapeutics against MM and other malignancies [3]. In this regard, we have demonstrated that miR-29b is a TS miRNA, whose reinforcement by synthetic miR-29b oligonucleotides triggers apoptosis both *in vitro* and *in vivo* in validated preclinical models of human MM [14, 20].

Genetic and epigenetic mechanisms may concur to downregulate miR-29b in human cancer [18]. Among epigenetic abnormalities, we previously demonstrated that the class II histone deacetylase HDAC4 exerts a negative control on miR-29b, and its silencing increases acetylation at miR-29a/b-1 promoter region thus triggering miR-29b expression in MM cells [19]. So far, the contribution of other MM-related epigenetic modifications to miR-29b regulation, such as histone methylation, has not been addressed. Noteworthy, we have here demonstrated, for the first time at our knowledge, a negative association between the H3K27 methyltransferase EZH2 and miR-29b in MM.

EZH2 is a histone methyltransferase member of the polycomb repressive complex PRC2, which also includes EED, SUZ12, RbAp48 and other accessory proteins [23, 33, 34]. The SET domain of EZH2 represents the catalytic subunit which catalyzes the tri-methylation of H3K27, a histone post-translational modification associated with the repression of gene expression [35]. Importantly, EZH2 has been largely involved in B-cell pathophysiology, as demonstrated by evidence that, in germinal centers, high EZH2 levels trigger B cell expansion, while EZH2-activating mutations may promote development of diffuse large B-cell and follicular lymphomas [36, 37]. EZH2 mRNA expression increases during progression from monoclonal gammopathy of undetermined significance (MGUS) through smoldering MM to overt MM [38, 39], and high EZH2 mRNA expression in newly-diagnosed MM patients associates with poor outcomes and high-risk clinical features [26]. All these findings underscore a relevant role of EZH2 in MM pathogenesis. Interestingly, EZH2 and its homolog EZH1 are promising targets for therapeutic intervention in MM, since small molecule inhibitors of EZH1/2 trigger cell cycle arrest and apoptosis in MM cell lines and primary CD138^+^ cells [25, 26, 40], and stem cells [41] as consequence of upregulation of H3K27me3-silenced genes, including the CDK inhibitors CDKN1A/p21 and CDKN2B/p15.

By interrogating GEP and miRNA datasets, we found that miR-29b and EZH2 mRNA levels inversely correlated. This finding prompted us to investigate whether EZH2 might regulate miR-29b. In this regard, we here provide formal proof that EZH2 exerts a negative control on miR-29b, since its genetic or pharmacological inhibition triggers miR-29b upregulation. Mechanistically, we here demonstrate that EZH2 binds the miR-29a/b-1 promoter, and H3K27me3 repressive marks at miR-29a/b-1 promoter may contribute to miR-29b silencing, since reduced H3K27me3 at miR-29a/b-1 promoter accompanies miR-29b upregulation upon EZH2 pharmacological blockade. We also show that EZH2 inhibition triggers a functionally active miR-29b, as demonstrated by downregulation of validated miR-29b targets in MM cells transfected with EZH2-targeting siRNAs or treated with small molecule EZH2 inhibitors.

**Figure 6:**
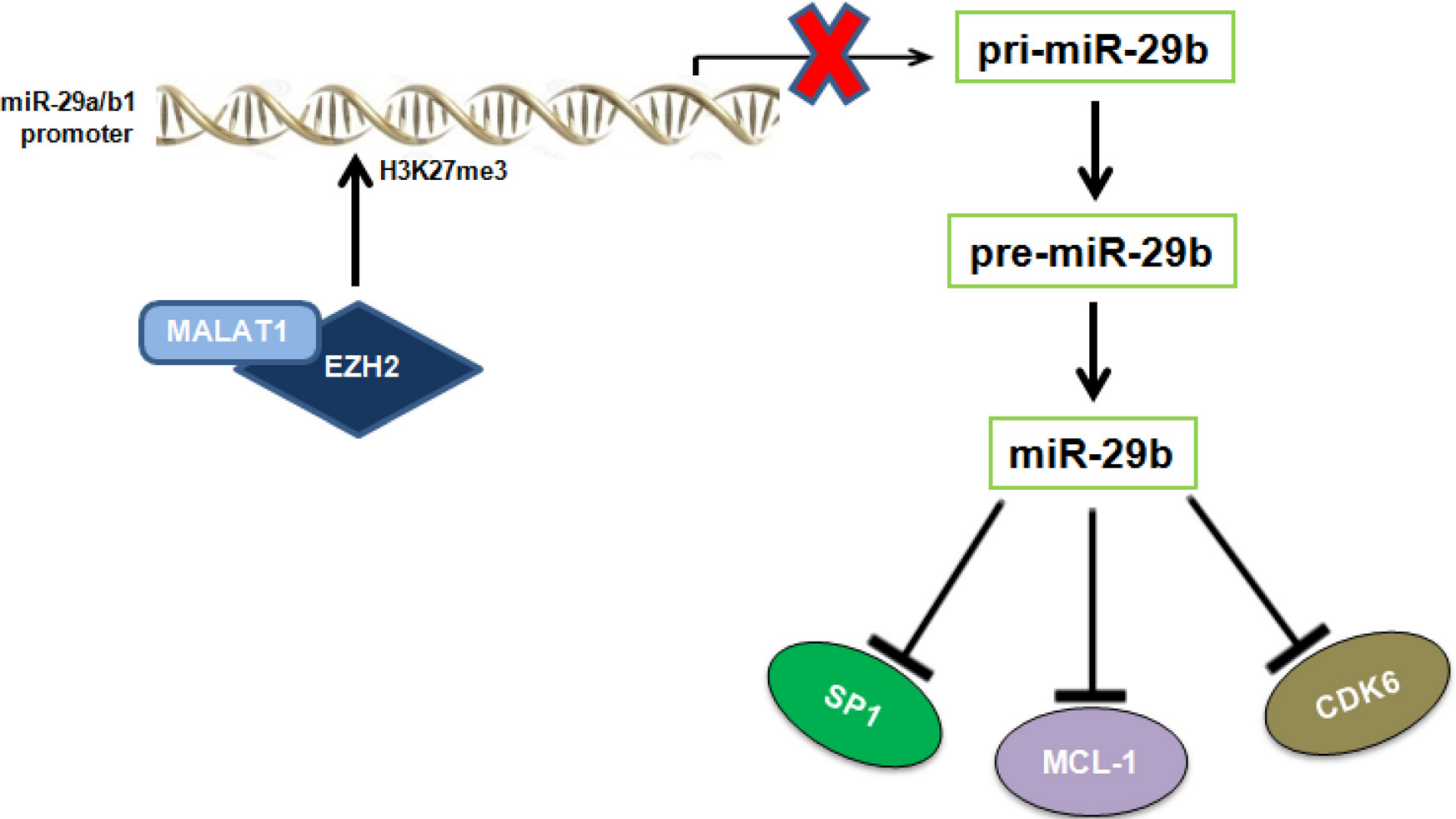
Graphic overview of the inhibitory effect played by EZH2 and MALAT1 on miR-29b expression The cartoon shows that MALAT1 and EZH2 negatively regulates miR-29b expression by increasing H3K27me3 of miR-29a/b-1 promoter, thus affecting levels of miR-29b oncogenic targets SP1, MCL-1 and CDK6.

Recent evidence has suggested that the lncRNA MALAT1 interacts with members of the PRC2 complex and positively modulates the H3K27me3 activity of EZH2. LncRNAs represent more than half of the mammalian non-coding transcriptome, and are involved in different biological processes, such as transcriptional regulation, maintenance of genomic integrity, X-chromosome inactivation, genomic imprinting, cell differentiation, and development [32, 42]. MALAT1 acts as oncogenic lncRNA in a wide variety of solid and hematological malignancies, including osteosarcoma [28], renal cell carcinoma [29], colorectal [43], prostate cancer [30], esophageal carcinoma [44], T, NK [45] and mantle cell lymphomas [46]. Recent reports indicate that high MALAT1 expression levels predict poor prognosis of cancer patients [28, 29] and MALAT1 knock-down blocks epithelial to mesenchymal transition and triggers cell cycle arrest and apoptosis, associated to reduced binding of EZH2 to its target loci and consequent upregulation of EZH2-repressed genes [28, 29, 47]. We recently carried out the first lncRNA profiling of PC malignancies, which identified several lncRNAs deregulated in tumor samples compared to normal controls; among these, upregulation of MALAT1 was associated with activation of pathways involved in cell cycle regulation [48]. In line with its acknowledged role as EZH2 positive modulator, silencing of MALAT1 by ASOs mimicked the molecular effects produced by EZH2 inhibition, as shown by: *i*) miR-29b upregulation, *ii*) reduced H3K27me3 at miR-29a/b-1 promoter, and *iii*) downregulation of miR-29b target genes upon MALAT1 inhibition. The effects of MALAT1 and EZH2 on miR-29b expression are summarized in Figure 6.

Finally, the findings that miR-29b blockade dramatically impairs the anti-MM activity of DZNep, GSK343 and EPZ005687 disclose relevant implications for the design of targeted therapeutic approaches against MM and other malignancies, in which the tumor suppressive role of miR-29b is well acknowledged. Given that miR-29b seems to be an important effector of the anti-MM activity of small molecule EZH2 inhibitors, it is reasonable that the design of anti-tumor strategies targeting EZH2 should take into account the miR-29b status of tumor cells, and that upregulation of miR-29b after treatment may be likely predictive of response to these drugs.

In conclusion, our findings widen the spectrum of epigenetic abnormalities inducing downregulation of miR-29b in MM, and provide the molecular basis to rationally develop tailored epigenetic therapies against this still fatal malignancy.

## MATERIALS AND METHODS

### Cell lines, transfection and reagents

JJN3 MM cell line was purchased from DSMZ and cultured in DMEM supplemented with 20% FBS and 1% P/S (Gibco^®^, Life Technologies), while AMO-BZB MM cell line was provided by Dr. C. Driessen (University of Tubingen, Germany), and cultured in RPMI-1640 (Gibco^®^, Life Technologies), supplemented with 10% FBS, 1% P/S (Gibco^®^, Life Technologies), at 37°C in a 5% CO_2_ atmosphere. JJN3 and AMO-BZB transfection was performed by the Neon^®^ Transfection System (Life Technologies) as previously described [49], with the following electroporation protocol: 2 pulses, 1050 V, 30 milliseconds. Silencer^®^ Select siRNA for EZH2 (siEZH2) and Silencer^®^ Select siRNA control (SCR) were purchased from Life Technologies and used at final concentration of 100 nM. MALAT1 synthetic inhibitors (MALAT1i) LNA™ longRNA GapmeRs were purchased by Exiqon (Vedbaek, Denmark). MALAT1i sequence was ACATTGCCTCTTCATT; a mismatch LNA™ longRNA GapmeR (named g CNT: GCTCCCTTCAATCCAA) was used as negative control. 3-Deazaneplanocin A (DZNep) was purchased by Sigma Aldrich and dissolved in DMSO; EPZ005687 and GSK343 were purchased from Selleckchem and dissolved in DMSO.

### Reverse transcription (RT) and quantitative real-time amplification (qRT-PCR)

Total RNA was extracted from cells using TRIzol^®^ reagent (Gibco, Life Technologies, Carlsbad, CA), following the manufacturer’s instructions. The RNA quantity and quality was assessed through NanoDrop^®^ (ND-1000 Spectrophotometer). To evaluate gene expression levels, 1000 ng of total RNA were reverse transcribed to cDNA using the “High Capacity cDNA Reverse Transcription Kit” (Applied Biosystems, Carlsbad, CA). The single-tube TaqMan assays (Applied Biosystems, Carlsbad, CA) were used to detect and quantify genes: EZH2 (Hs01016789_m1), MALAT1 (Hs00273907_s1) according to the manufacturer’s instructions, using Viia 7 Dx multicolor detection system (Applied Biosystems, Carlsbad, CA). The obtained Threshold Cycle (CT) values were normalized on GAPDH (Hs03929097_g1). The single-tube TaqMan miRNA assay (Applied Biosystems, Assay id 000413) was used to detect and quantify mature miR-29b expression, that was normalized on RNU44 (Applied Biosystems, Assay id 001094). Comparative real-time polymerase chain-reaction (RT-PCR) was carried out in triplicate, including no-template controls. Relative expression was calculated using the comparative cross threshold (Ct) method.

### Expression vectors

pEZ-M06-EZH2 plasmid containing EZH2 cDNA and the corresponding empty vector were from Genecopoeia (GeneCopeia, Rockville, MD, USA). MALAT1 cDNA in pCMV-SPORT6 (Dharmacon GE) was subcloned by the Genomics facility of Biogem IRGS (Ariano Irpino, Italy) using PmeI and BamH1 restriction sites, in a modified pGREENpuro lentiviral vector (System Biosciences) where the H1 promoter was replaced with the CMV promoter.

### Western blotting

SDS-PAGE and Western Blotting (WB) were carried out according to standard protocols [50, 51]. Briefly, cells were lysed and whole cells lysates (∼50 μg per lane) were separated using 4–12% Novex Bis-Tris SDS-acrylamide gels (Invitrogen) and electro-transferred on nitrocellulose membranes (Bio-Rad). Then, nitrocellulose membranes were blocked with milk and probed over-night with primary antibodies at 4°C, then membranes were washed 3 times in PBS-Tween, incubated with a secondary antibody conjugated with horseradish peroxidase for 2 hours at RT. Chemiluminescence was detected using Western Blotting Luminol Reagent (Santa Cruz, Dallas, TX, USA). Signal intensity was quantified with the Quantity One Analyzing System (Bio-Rad). Antibodies used were the following: EZH2 (cat#5246S), CDK6 (cat#3136S) and MCL1 (cat#5453S) were from Cell Signaling, while SP1 (sc-14027), ACTIN (sc-1616) and GAPDH (sc-25778) were from Santa Cruz.

### Chromatin immunoprecipitation

For chromatin immunoprecipitation (ChIP) experiments, the ChIP Assay Kit (Pierce Agarose ChIP Thermo Fisher Scientific) was used. Cells (1.5 × 10^7^) were crosslinked in 1% formaldehyde, lysed, and sheared by sonication using the Bioruptor Plus (Diagenode), for 10 cycles (each of 30 seconds) on a cold block, with 90 seconds intervals of cooling. Chromatin was divided into equal amounts for immunoprecipitation with the histone H3 (tri-methyl K27) antibody (Abcam, ChIP Grade ab6002), EZH2 (Abcam, ChIP Grade ab3748) or rabbit IgG as negative control (Santa Cruz Biotechnology). Chromatin extracts were incubated on a rotator with 20 μl of ChIP Grade Protein A/G Plus Agarose for 3 h at 4°C. Bound agarose beads were harvested by centrifugation (12.000rpm, 15 seconds) and washed; precipitated protein-DNA complexes were eluted from the washed beads and incubated twice at 65°C for 1.5 h with NaCl and Proteinase K to revert cross-links. Purified DNA was subjected to qRT-PCR using GoTaq qPCR Master Mix (Promega). Primer sequences for qPCR were as follows: miR-29a-b1 promoter: Fw 5′-3′: CATGCCTGTAGTGAGGCTGA; Rev 5′-3′: TCCTGAGTAGCTGG GATTGC.

### Virus generation and infection of MM cells

293Ta cells were co-transfected with 10 μg of lentiviral expression vector or the corresponding empty vector, 10 μg of pCMV-VSVG, and 4 μg of ∆8.9 plasmids as previously reported [14]; supernatants were collected 48 hours after 293Ta transfection, and used to transduce MM cells by a single round of 15 hours of infection. MM cells were then selected in medium containing 0.5 μg/mL puromycin (Sigma Aldrich).

### Analysis of cell viability and proliferation

Cell viability and proliferation were analyzed by Cell Titer Glo (Promega) and Trypan blue exclusion (Sigma Aldrich), respectively, as described [50].

### Statistical analysis

Each experiment was performed at least 3 times, and values reported as means +/− SD. Comparisons between groups were made with Student *t* test, while statistical significance of differences among multiple groups was calculated by GraphPad software (www.graphpad.com). Only results with *P* < 0.05 were accepted as statistically significant.
